# Application of a methodological framework for the development and multicenter validation of reliable artificial intelligence in embryo evaluation

**DOI:** 10.1186/s12958-025-01351-w

**Published:** 2025-01-31

**Authors:** D. Gilboa, Akhil Garg, M. Shapiro, M. Meseguer, Y. Amar, N. Lustgarten, N. Desai, T. Shavit, V. Silva, A. Papatheodorou, A. Chatziparasidou, S. Angras, J. H. Lee, L. Thiel, C. L. Curchoe, Y. Tauber, D. S. Seidman

**Affiliations:** 1 AIVF Ltd, Tel Aviv, Israel; 2 IVIRMA Valencia, Valencia, Spain; 3 Health Research Institute La Fe, Valencia, Spain; 4 Department of Obstetrics and Gynecology, Division of Reproductive Endocrinology and Infertility, Women’s Health Institute, Cleveland Clinic, Beachwood, OH USA; 5 In Vitro Fertilization (IVF) Unit, Assuta Ramat HaHayal, Tel-Aviv, Israel; 6 Ferticentro - Centro de Estudos de Fertilidade, Coimbra, Portugal; 7 Procriar - Clínica de Obstetrícia e Medicina da Reprodução do Porto, Porto, Portugal; 8 Embryolab Fertility Clinic, Thessaloniki, Greece; 9 FIRST IVF Clinic, Clane, Ireland; 10 Maria Fertility Hospital, Goyang, Republic of Korea; 11 Praxis Dres.med. Göhring, Tübingen, Germany; 12 Art Compass, an AIVF Technology, Newport Beach, CA USA

**Keywords:** Artificial intelligence, Time lapse imaging, Embryo ranking, Machine learning

## Abstract

**Background:**

Artificial intelligence (AI) models analyzing embryo time-lapse images have been developed to predict the likelihood of pregnancy following in vitro fertilization (IVF). However, limited research exists on methods ensuring AI consistency and reliability in clinical settings during its development and validation process. We present a methodology for developing and validating an AI model across multiple datasets to demonstrate reliable performance in evaluating blastocyst-stage embryos.

**Methods:**

This multicenter analysis utilizes time-lapse images, pregnancy outcomes, and morphologic annotations from embryos collected at 10 IVF clinics across 9 countries between 2018 and 2022. The four-step methodology for developing and evaluating the AI model include: (I) curating annotated datasets that represent the intended clinical use case; (II) developing and optimizing the AI model; (III) evaluating the AI’s performance by assessing its discriminative power and associations with pregnancy probability across variable data; and (IV) ensuring interpretability and explainability by correlating AI scores with relevant morphologic features of embryo quality. Three datasets were used: the training and validation dataset (*n* = 16,935 embryos), the blind test dataset (*n* = 1,708 embryos; 3 clinics), and the independent dataset (*n* = 7,445 embryos; 7 clinics) derived from previously unseen clinic cohorts.

**Results:**

The AI was designed as a deep learning classifier ranking embryos by score according to their likelihood of clinical pregnancy. Higher AI score brackets were associated with increased fetal heartbeat (FH) likelihood across all evaluated datasets, showing a trend of increasing odds ratios (OR). The highest OR was observed in the top G4 bracket (test dataset G4 score ≥ 7.5: OR 3.84; independent dataset G4 score ≥ 7.5: OR 4.01), while the lowest was in the G1 bracket (test dataset G1 score < 4.0: OR 0.40; independent dataset G1 score < 4.0: OR 0.45). AI score brackets G2, G3, and G4 displayed OR values above 1.0 (*P* < 0.05), indicating linear associations with FH likelihood. Average AI scores were consistently higher for FH-positive than for FH-negative embryos within each age subgroup. Positive correlations were also observed between AI scores and key morphologic parameters used to predict embryo quality.

**Conclusions:**

Strong AI performance across multiple datasets demonstrates the value of our four-step methodology in developing and validating the AI as a reliable adjunct to embryo evaluation.

**Supplementary Information:**

The online version contains supplementary material available at 10.1186/s12958-025-01351-w.

## Introduction

Top-quality, euploid embryo selection is a central dogma of current human IVF practice [1, 2]. The development of “embryo morphokinetics” goes beyond simple morphological assessment by adding a longitudinal, “time to milestone” assessment factor. The ability to build accurate predictive models via morphokinetic data [3] has been enhanced with the introduction of time-lapse imaging (TLI) and incubation systems [4]. In the IVF laboratory, embryo activity can now be monitored throughout its in vitro development inside the TLI, increasing embryologist agreement in assessment [5, 6] and clinical decision making [7]. Measurable differences in embryo development, kinetics, and morphology can be quantifiably assessed, and artificial neural networks, trained on these features, can make accurate predictions, as shown in a handful of studies. To date, multiple artificial neural networks (ANNs), a key method in artificial intelligence (AI), have been developed, and pre-clinical, retrospective, non-randomized studies have been used to validate their performance across several applications in medically assisted reproduction. These include gamete assessment [8, 9], automated annotation of key embryo development events [6, 10], viability prediction [11], implantation prediction [12–14], ploidy prediction [15–19], diagnostic applications [20], and clinical decision-making support [21, 22].

Validation studies highlight the strengths and limitations of the AI predictive model and identify potential issues that must be addressed before advancing to clinical trials or real-time applications. To effectively validate an AI model as reliable and consistent, several key aspects should be presented, such as well-curated and balanced datasets, model robustness, and performance repeatability [23, 24]. The AI should demonstrate consistent performance despite variations in clinical practice, such as embryo manipulation techniques and clinician expertise, as well as differences in patient demographics and data imbalances, where certain patient or embryo subgroups (e.g., based on quality or genetic profile) may be underrepresented. This is critical for ensuring the efficacy and reliability of new technologies or models before they are implemented in a broader clinical context [30, 31].

Furthermore, if the intended use of AI for embryo quality assessment is to assist an embryologist in selecting the best embryo for transfer from a patient cohort containing multiple embryos in each IVF cycle, the AI-driven score should reliably correlate with positive clinical outcomes. Several reviews succinctly present the current state of the art [25–29].

The primary objectives of this study are to evaluate the performance of the AIVF Day-5 AI model (AIVF, Tel Aviv, Israel) as accurate and reliable, discern the AI’s capacity to handle variations and complexities inherent in real-world data, and establish a performance foundation for future prospective validation in clinical settings. To achieve these objectives, this study employs a four-step methodology: (I) curating diverse, annotated datasets that represent the clinical use case; (II) developing and optimizing the AI model; (III) evaluating the AI’s consistent and reliable performance across varying datasets; IV) ensuring clinical relevance by visualizing correlations with conventional embryo quality features.

## Materials and methods

### Data curation and annotation

All collected data were de-identified and retrospectively collected prior to data analysis. The de-identified datasets curated and utilized for this study include the training and validation dataset, the blind test dataset and the independently collected real-world dataset. The full training and validation dataset (*n =* 16,935 embryos; 3 clinics) was used for AI model development and optimization. This dataset consisted of 9,810 annotated time-lapse videos of embryos transferred on day 5 with known pregnancy outcome data (positive/negative fetal heartbeat at first transvaginal ultrasound after transfer) in addition to 7,125 embryos that were manually discarded for poor quality or aneuploidy. The blind test dataset (*n* = 1,708 embryos; 3 clinics) and the independently collected real-world dataset (*n* = 7,445 embryos; 7 clinics), referred to as the independent dataset, were used to assess AI performance and consistency across multiple clinical settings and varying data types. The test data overlapped at the clinic level with the data used for AI model training and validation but remained independent at the patient and treatment levels. Meanwhile, the independent dataset had no overlap at the clinic, patient, or treatment level with any training or test data. This dataset represents new demographic and clinical profiles that were not previously encountered by the AI model. No specific selection criteria were applied to participating clinics, ensuring that the real-world demographic composition of each clinic was preserved. Embryos resulting from fresh autologous oocyte retrievals, oocyte donors, fresh transfers, and vitrification-warming cycles, with or without PGT-A, were included. All embryos were derived from IVF/ICSI cycles conducted between 2018 and 2022.

Each clinic followed its standard operating protocols for ovarian stimulation, oocyte retrieval, fertilization, embryo culture, observation, and vitrification. All normally fertilized zygotes with two pronuclei were cultured until the blastocyst (Day 5) stage. All embryos were cultured and observed in EmbryoScopeTM, EmbryoScopeTM+, or EmbryoScopeTM Flex incubators (Vitrolife A/S, Aarhus, Denmark). The culture dish was undisturbed, and not removed from the incubator for media refresh or assisted hatching. The embryo transfer procedure was performed according to each clinic’s standard operating practice.

Embryo image acquisition via the TLI incubator software was performed according to the manufacturer’s instructions. For the Embryoscope platform, a full time-lapse video sequence was generated from static two-dimensional images captured at regular intervals of 5–15 min in multiple focal planes (7 focal planes for EmbryoscopeTM; 11 focal planes for EmbryoScopeTM+, or EmbryoScopeTM Flex). The AI model only calculates a score for embryos cultured until at least 105 h after insemination, therefore, this study only included embryos that reached this time of development in vitro. Associated morphology annotations, oocyte source (autologous or donor oocyte recipient), patient age, and clinical pregnancy outcomes were collected from each participating clinic from the electronic medical record (EMR), when available.

### AI model development and optimization

The AI was built as a deep learning-based classifier trained to rank embryos by score (1.0-9.9) according to their likelihood of ongoing clinical pregnancy, defined as fetal heartbeat (FH) at transvaginal ultrasound. The AI was developed as an outcome-based model using embryo time-lapse sequence data, without the need for user input or the extraction of discrete morphological and morphokinetic variables. It was trained exclusively on embryos removed from the time-lapse incubator after 105 h post-insemination (hpi).

A description of the full training and validation dataset used for model development and optimization is presented in Supplemental Table 1. The data were randomly partitioned into a two-part 85:15% train/validation data split for training iterations and hyperparameters optimization. This was done evenly for all embryos across all treatments and clinics. The models were trained using Amazon Web Services (AWS) infrastructure, specifically on g4dn.xlarge instances equipped with an NVIDIA T4 GPU (16 GiB) and 16 GiB of RAM.

The deep learning classifier was developed as a pipeline of models (Supplemental Fig. 2). The core classifier model in the pipeline has an overall neural network structure of a Resnet50 backbone with two separate binary classification heads: one for positive/negative fetal heartbeat (FH+/FH-) classification, and one for discarded/usable embryo classification (based on morphologic quality and/or developmental competence by Day 5). Each head consists of a pair of fully connected layers, followed by a dropout layer and a sigmoid activation function layer. Notably, discarded embryos were included in the training/validation dataset and pseudo labeled as FH- to ensure that the scores remained within a reasonable distribution across the entire range of embryo qualities, including those with poor development or morphology. The use of two discrete classification heads ensured that pseudo labeled FH- embryos were differentiated from true FH- embryos.

Single image frames were extracted from the time-lapse sequence and used as core inputs for the model. The model sampled all image frames within the temporal window of the blastocyst stage of development. Only frames from the central focal plane (z = 0 μm) were selected, as these images were typically the sharpest at this focal point. Before feeding the image into the network, each image was preprocessed by cropping it around its center point, as detected using a pre-trained segmentation network (UNet), and then resized to a resolution of 224 × 224 pixels. The segmentation network, based on the UNet architecture, was trained using Keras to create a binary mask for the embryos. The training database consisted of 2,000 annotated frames, with 90% used for training and 10% for validation, covering all stages of embryo development, from the oocyte stage to the hatching blastocyst. The frames were annotated by drawing polygons around the embryo cells, and the annotations were validated internally before use. The model achieved a Dice coefficient of 0.972 [95% CI: 0.95–0.987]. The core model was trained using a batch size of 24; each batch was sampled to include 50%, 25%, 25% FH+, FH-, and discarded embryos, respectively.

For model inference, the time lapse sequence data was tested using a dedicated deep learning-based classifier to detect the presence and time of blastulation. This blastulation model was trained as a classifier using a ResNet-50 backbone to distinguish between frames captured before the start of blastulation (annotated tSB) and frames captured after full blastocyst formation (annotated TB). This model was trained on a dataset of 48,168 frames extracted from 500 embryos, which were split into 80% for training and 20% for validation. The model achieved an ROC-AUC of 0.999 [95% CI: 0.9998-1.0000].

The core classifier model was then applied to all sampled frames of the blastocyst-stage embryo, generating a vector of fluctuating scores. This vector was input into a heuristic model that considered the time of blastulation (as determined by the blastulation model) and the rate of blastocyst expansion. Rate of expansion was measured by calculating the blastocyst area using the segmentation network at predefined time-points after blastulation. Using selective decision rules, these parameters enabled the automatic detection of embryo manipulation events such as artificial collapse and assisted hatching. The vector of scores was smoothed to generate a final scalar rating for each embryo (ranging from 1.0 to 9.9), which correlates with the likelihood of FH. Embryos that did not reach blastulation were assigned the minimum score (1.0). After the training and validation phases, the model pipeline remains static.

### Study analysis to evaluate consistent and reliable AI performance

The blind test dataset and the independent dataset not involved in model development were evaluated to assess overall performance and consistency across variable data. Embryos missing patient age or oocyte source information (either autologous or donor oocyte recipient) were excluded from analysis. Overall, 1,199 embryos were excluded, for a final embryo count of 1,708 in the test dataset and 6,246 in the independent dataset. Standard statistical methods were used for these validations. Data for continuous variables were expressed as mean ± SD. Fisher’s exact or chi-squared tests were performed for categorical variables. Logistic regression analyses were performed to confirm significant associations and calculate the odds-ratios (OR) between comparisons. A *p*-value < 0.05 was considered significant. All statistical analyses were performed with the R software version 4.3.1. Classification performance of the AI model was evaluated using the pROC package for estimating the ROC (Receiver Operating Characteristic) curve and AUC (Area Under Curve).

### Correlation with conventional annotations

The clinical relevance of the model was evaluated by visualizing correlations to conventional embryo quality features, ensuring alignment with the gold standard for embryo evaluation in clinical practice. For this, we included all embryos in the test and independent datasets with known morphology annotations (*n =* 7,092 embryos) manually assigned onsite at each participating clinic by expert embryologist 105 h or more after insemination, according to Gardner or ASEBIR embryo quality classification schemes [32–34]. The quality parameters, as defined by both schemes, include degree of blastocyst expansion, integrity of the inner cell mass (ICM), and trophectoderm (TE). For conformity, Gardner quality scores were mapped to the ASEBIR score system prior to analysis using an automated Python script according to the morphology classification system described by the Embryology Interest Group of the Association for the Study of Reproductive Biology [33, 34]. Correlations between AI scores and the morphology of the inner cell mass (ICM), trophectoderm (TE) and overall ASEBIR embryo quality were analyzed for all transferred and non-transferred annotated embryos.

To evaluate the correlations between AI scores and ongoing clinical pregnancy outcome, we limited the data to embryos with known fetal heartbeat (FH) data after single embryo transfer *(n =* 1,959 embryos). Data were analyzed and expressed using the same methods and software described above.

## Results

### Data curation and annotation

Supplemental Table 2 describes the test and independent datasets used for AI validation in this manuscript. The breakdown of the clinical outcome data was as follows: within the blind test dataset, 1,070 blastocyst-stage embryos were transferred (single embryo transfer *n* = 997; double embryo transfer *n =* 64; triple embryo transfer *n =* 9). Within the independent dataset, 1,493 blastocyst-stage embryos were transferred (single embryo transfer *n =* 1,168; double embryo transfer *n =* 316). In total, 2,563 blastocyst-stage embryos were transferred (single embryo transfer *n =* 2,165; double embryo transfer *n =* 380; triple embryo transfer *n =* 9). Since the contribution of multiple embryo transfer cycles in the datasets was not significant, only clinical outcomes derived from single embryo transfer (SET) cycles were considered for clinical pregnancy outcome analysis, as described in Supplemental Table 3. Of the 2,165 SET embryos transferred across both datasets, known pregnancy outcomes were recorded for 1,959 embryos; 975 embryos were positive for clinical pregnancy; 984 embryos were negative for clinical pregnancy.

The breakdown of morphologic annotation data was as follows: 1,358 embryos in the test dataset and 5,734 embryos in the independent dataset were morphologically annotated, respectively. In total, 7,092 embryos had morphological annotations available for evaluation (Supplemental Table 2).

The mean maternal age (uterus age) across both datasets was comparable (38.76 ± 5.21 in blind test dataset; 38.76 ± 5.21 in the independent dataset). The mean oocyte age (age of autologous or oocyte donor patient) was different between datasets (30.20 ± 6.96 in blind test dataset; 35.79 ± 4.12 in the independent dataset). This difference accounts for the inclusion of donor oocyte recipient cycles in the data; In the test dataset, 415/997 (41.6%) of transferred embryos were derived from donor cycles; in the independent dataset, 232/1,168 (19.9%) of transferred embryos were derived from donor cycles (Supplemental Table 3). This reflects the inherent variability in patient demographics found in multicenter data and underscores the importance of comprehensive subgroup comparisons in the AI’s validation. Accordingly, data were stratified by maternal age, oocyte age, and oocyte source (autologous or donor oocyte recipient) in both datasets. These stratifications are presented in Table 1 for the test dataset and Table 2 for the independent dataset.

**Table 1:** Test dataset characteristics. Double stratification of data by age (< 35; 35–37, 38–40, 41–43, > 43 years) and by different subgroups of maternal (uterus) age, oocyte age, and oocyte source (autologous and donor oocyte recipient). Odds ratio (OR) and *P*-values for the associations between each subgroup and fetal heartbeat (FH) rate is shown. Mean AI score per age category is also shown. All transferred embryos in this analysis had known FH clinical outcomes

	Characteristics	Age group (years)				
		< 35	35–37	38–40	41–43	> 43
Maternal (Uterus) Age	Transferred embryos (n)	285	196	180	183	142
	FH + embryos (n)	135	103	89	86	79
	FH rate (%) [95% CI]	47.36 [41.5–53.2]	52.55 [45.5–59.60]	49.44 [42.10–56.80]	46.99 [39.70–54.30]	55.63 [47.40–63.90]
	Age (mean ± SD, years)	31.3 ± 2.76	36.0 ± 0.81	39.2 ± 0.78	41.9 ± 0.83	45.7 ± 1.71
	OR [95% CI]	0.90 [0.71–1.14]	1.23 [0.86–1.77]	1.09 [0.75–1.58]	0.99 [0.68–1.42]	1.39 [0.93–2.09]
	*P*-value	0.374	0.264	0.662	0.937	0.108
	AI score (mean ± SD)	6.24 ± 2.45	5.93 ± 2.43	5.98 ± 2.29	5.82 ± 2.66	6.38 ± 2.25
Oocyte Age	Transferred embryos (n)	669	155	84	59	20
	FH + embryos (n)	369	81	28	11	3
	FH rate (%) [95% CI]	55.15 [51.40–58.90]	52.25 [44.30–60.20]	33.33 [23.00-43.60]	18.64 [8.41–28.90]	15.0 [0.0-32.10]
	Age (mean ± SD, years)	27.6 ± 4.78	35.9 ± 0.82	39.0 ± 0.79	41.7 ± 0.81	44.8 ± 0.76
	OR [95% CI]	1.230 [1.056–1.432]	0.890 [0.627–1.263]	0.407 [0.252–0.656]	0.186 [0.095–0.360]	0.143 [0.042–0.494]
	*P*-value	0.007	0.513	< 0.001	< 0.001	0.002
	AI score (mean ± SD)	6.50 ± 2.22	5.82 ± 2.45	4.99 ± 2.43	4.06 ± 2.79	4.33 ± 2.63
Autologous IVF Cycles	Transferred embryos (n)	254	155	84	59	20
	FH + embryos (n)	113	81	28	11	3
	FH rate (%) [95% CI]	44.48 [38.30–50.60]	52.25 [44.30–60.20]	33.33 [23.00-43.60]	18.64 [8.41–28.90]	15.0 [0.0-32.10]
	Age (mean ± SD, years)	31.2 ± 2.82	35.9 ± 0.82	39.0 ± 0.79	41.7 ± 0.81	44.8 ± 0.76
	OR [95% CI]	0.80 [0.63–1.03]	1.37 [0.92–2.04]	0.62 [0.37–1.05]	0.29 [0.14–0.57]	0.22 [0.06–0.77]
	*P*-value	0.079	0.127	0.073	< 0.001	0.017
	AI score (mean ± SD)	6.12 ± 2.51	5.82 ± 2.45	4.99 ± 2.43	4.06 ± 2.79	4.33 ± 2.63
Donor Oocyte Recipient IVF Cycles	Transferred embryos (n)	31	41	96	124	122
	FH + embryos (n)	22	22	61	75	76
	FH rate (%) [95% CI]	70.96 [54.00-87.90]	53.65 [37.70–69.60]	63.54 [53.70–73.30]	60.48 [51.80–69.20]	62.29 [53.60–71.00]
	Oocyte age (mean ± SD, years)	25.9 ± 3.75	25.8 ± 4.60	25.2 ± 4.40	25.0 ± 4.26	25.6 ± 4.45
	Maternal age (mean ± SD)	31.9 ± 2.08	36.2 ± 0.759	39.3 ± 0.771	42.0 ± 0.836	45.9 ± 1.78
	OR [95% CI]	2.44 [1.13–5.31]	0.47 [0.18–1.27]	0.713 [0.30–1.72]	0.62 [0.27–1.47]	0.68 [0.29–1.59]
	*P*-value	0.023	0.138	0.451	0.283	0.370
	AI score (mean ± SD)	7.25 ± 1.56	6.34 ± 2.34	6.86 ± 1.74	6.66 ± 2.14	6.72 ± 2.00

**Table 2:** Independent dataset characteristics. Double stratification of data by age (< 35; 35–37, 38–40, 41–43, > 43 years), and by different subgroups of maternal (uterus) age, oocyte age, and oocyte source (autologous and donor oocyte recipient). Odds ratio (OR) and *P*-values for the associations between each subgroup and fetal heartbeat (FH) rate is shown. Mean AI score per age category is also shown. All transferred embryos in this analysis had known FH clinical outcomes

	Characteristics	Age group (years)				
		< 35	35–37	38–40	41–43	> 43
Maternal (Uterus) Age	Transferred embryos (n)	408	287	235	140	98
	known transfer outcomes (n)	337	238	195	119	73
	FH + embryos (n)	175	123	84	54	43
	FH rate (%) [95% CI]	51.92 [46.60–57.30]	51.68 [45.30–58.10]	43.07 [36.10–50.10]	45.37 [36.30–54.50]	58.90 [47.30–70.50]
	Age (mean ± SD, years)	31.8 ± 2.26	36.0 ± 0.80	39.0 ± 0.86	41.8 ± 0.77	45.8 ± 1.83
	OR [95% CI]	1.09 [0.88–1.30]	0.98 [0.70–1.36]	0.70 [0.49–0.99]	0.76 [0.50–1.16]	1.32 [0.79–2.20]
	*P*-value	0.443	0.884	0.045	0.208	0.291
	AI score (mean ± SD)	6.06 ± 2.67	5.98 ± 2.57	5.60 ± 2.73	5.74 ± 2.73	6.86 ± 1.98
Oocyte Age	Transferred embryos (n)	598	258	200	74	10
	Known transfer outcomes (n)	486	214	172	66	10
	FH + embryos (n)	273	111	68	20	1
	FH rate (%) [95% CI]	56.17 [51.70–60.60]	51.86 [45.10–58.60]	39.53 [32.20–46.90]	30.30 [18.90–41.70]	10.0 [0.0-32.60]
	Age (mean ± SD, years)	29.6 ± 4.41	36.0 ± 0.811	38.9 ± 0.877	41.7 ± 0.766	44.1 ± 0.316
	OR [95% CI]	1.29 [1.08–1.53]	0.83 [0.60–1.15]	0.507 [0.36–0.72]	0.34 [0.19–0.59]	0.09 [0.01–0.69]
	*P*-value	0.006	0.254	< 0.01	< 0.01	0.021
	AI score (mean ± SD)	6.43 ± 2.43	5.90 ± 2.64	5.39 ± 2.81	4.67 ± 2.92	5.43 ± 1.79
Autologous IVF Cycles	Transferred embryos (n)	394	258	200	74	10
	Known transfer outcomes (n)	329	214	172	66	10
	FH + embryos (n)	168	111	68	20	1
	FH rate (%) [95% CI]	51.06 [45.60–56.50]	51.86 [45.10–58.60]	39.53 [32.20–46.90]	30.30 [18.90–41.70]	10.0 [0.0-32.60]
	Age (mean ± SD)	31.8 ± 2.26	36.0 ± 0.811	38.9 ± 0.877	41.7 ± 0.766	44.1 ± 0.316
	OR [95% CI]	1.05 [0.85–1.31]	1.017 [0.72–1.44]	0.62 [0.43–0.91]	0.41 [0.24–0.73]	0.11 [0.01–0.84]
	*P*-value	0.657	0.924	0.013	0.002	0.034
	AI Score (mean ± SD)	6.04 ± 2.68	5.90 ± 2.64	5.39 ± 2.81	4.67 ± 2.92	5.43 ± 1.79
Donor Oocyte Recipient IVF Cycles	Transferred embryos (n)	14	29	35	66	88
	Known transfer outcomes (n)	8	24	23	53	63
	FH + embryos (n)	7	12	16	34	42
	FH rate (%) [95% CI]	87.5 [57.90–100.00]	50.0 [28.40–71.60]	69.56 [49.20–89.90]	64.15 [50.80–77.50]	66.67 [54.70–78.60]
	Oocyte age (mean ± SD, years)	27.6 ± 4.25	24.3 ± 4.43	25.5 ± 4.56	25.7 ± 4.74	25.0 ± 4.20
	Maternal age (mean ± SD)	31.9 ± 2.16	36.2 ± 0.689	39.3 ± 0.701	42.0 ± 0.754	46.0 ± 1.83
	OR [95% CI]	7.00 [0.86–56.90]	0.14 [0.02–1.35]	0.33 [0.03–3.18]	0.26 [0.03–2.24]	0.29 [0.03–2.48]
	*P*-value	0.068	0.089	0.335	0.217	0.255
	AI score (mean ± SD)	6.62 ± 2.38	6.73 ± 1.68	6.80 ± 1.85	6.93 ± 1.89	7.02 ± 1.95

### Study analysis to evaluate consistent and reliable AI performance

The AI model was used to retrospectively assign a score (1.0-9.9) to every embryo without influencing conventional embryo selection workflow or clinical decision-making. The results presented in this section evaluate the AI’s performance across heterogeneous datasets, establishing a foundation for its deployment in prospective clinical settings.

### Test dataset

Table 1 shows the test dataset sorted by different subgroups of maternal (uterus) age, oocyte age, and oocyte source. Age guidelines were chosen according to the Society for Assisted Reproductive Technology (SART) age categories (< 35, 35–37, 38–40, 41–43, and > 43 years old). FH rate and mean AI scores for all transferred embryos with known outcomes were calculated for each subgroup.

Stratification of data by oocyte age showed an inverse relationship between increasing oocyte age and decreasing FH rate. The OR values used to measure the association between oocyte age and FH rate were highest in the lowest oocyte age category (< 35: OR 1.230 [95% CI: 1.056–1.432]), and lowest in the highest oocyte age category ( > = 43: OR 0.143 [95% CI: 0.042–0.494]). *P*-values for OR were significant in every oocyte age category except for the 35–37 age group. Likewise, univariate analysis of variance showed significant differences in mean AI scores between the different age categories (*P* < 0.01), with an inverse relationship observed between increasing oocyte age and decreasing AI scores (*P* = 0.01).

Stratification of data by maternal (uterus) age showed a marginal, nonsignificant inverse relationship between increasing maternal age and decreasing FH rate (*P* = 0.73), with nonsignificant OR values observed for the associations between maternal age and FH rate in every age category (*P* > 0.1). Likewise, univariate analysis of variance showed a nonsignificant difference in mean AI scores between the different age categories (*P* = 0.16). These results are expected, given the high prevalence of donor-derived FH + embryos in this test dataset (256 out of 496 FH + embryos were donor-derived, which confounded the influence of maternal age on FH rates and AI scores).

### Independent dataset

Table 2 shows the independent dataset sorted by different subgroups of maternal age, oocyte age, and oocyte source. Stratification of data by oocyte age revealed an inverse relationship between oocyte age and FH rate, as observed in the test dataset. OR values for the association between oocyte age and FH rate were also highest in the lowest oocyte age category (< 35: 1.289 [95% CI: 1.077–1.543], and lowest in the highest oocyte age category ( > = 43: 0.086, [95% CI 0.011–0.686]). *P*-values for ORs were significant in every oocyte age category except for the 35–37 age group, which was consistent with the test dataset. A significant difference in mean AI scores between the different age categories was also observed (*P* < 0.01), with increasing oocyte age inversely correlated with AI scores (*P* = 0.01).

Stratification of the data by maternal age showed a nonsignificant inverse relationship between increasing maternal age and decreasing FH rate (*P* = 0.63). This relationship was consistent with that observed in the test dataset, due to the inclusion of donor-derived FH + embryos. In contrast to the test dataset, mean AI scores remained statistically different between the different maternal age categories in the independent dataset (*P* < 0.01).

### Discriminative AI performance

**Fig. 1:**
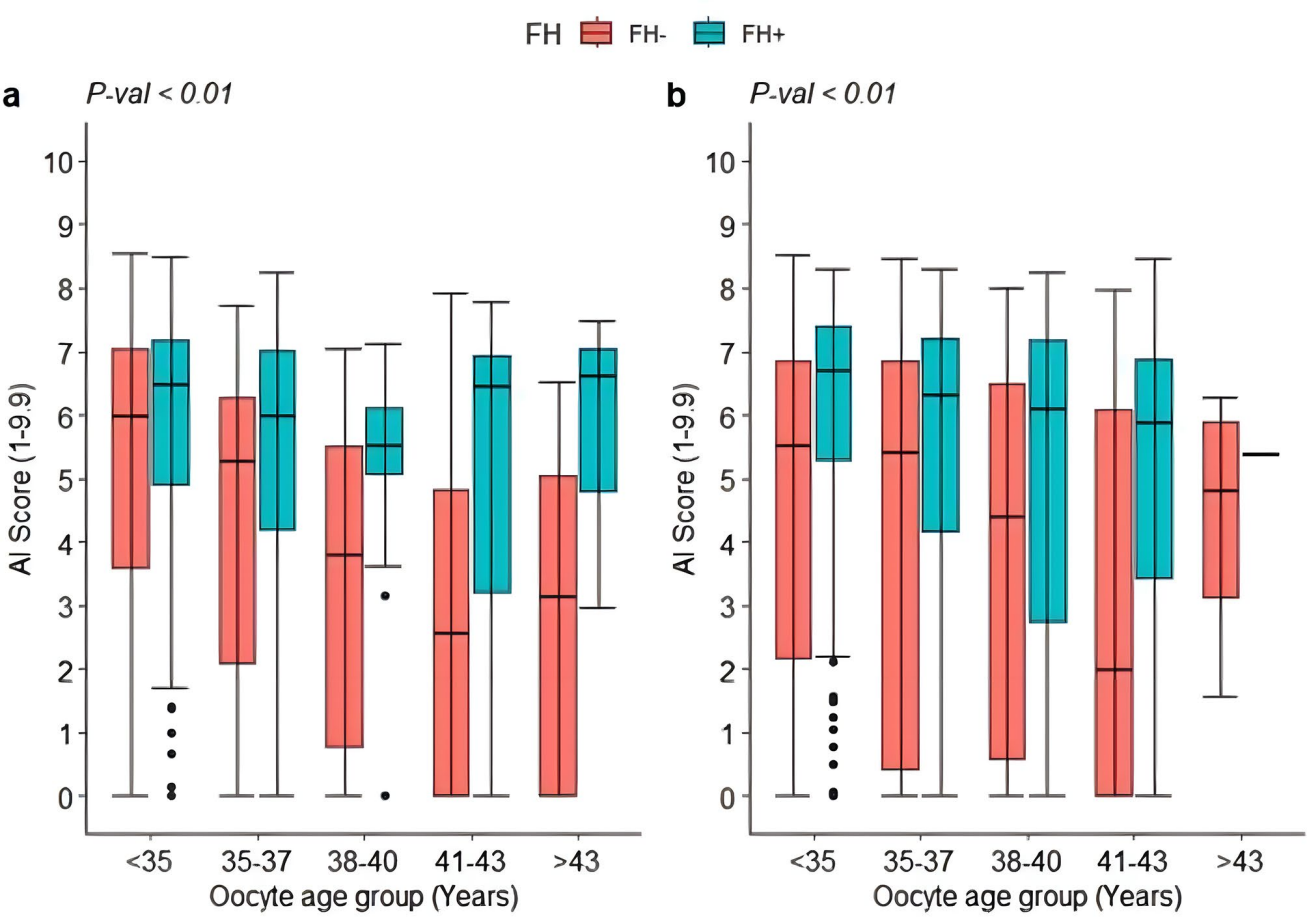
Evaluating the distribution of AI scores (ranging from 1-9.9) for fetal heartbeat positive (FH+) and fetal heartbeat negative (FH-) embryos per age group (oocyte age) in the test dataset (**a**) and independent dataset (**b**), respectively. Analysis of variance showed a significant difference in AI scores between age groups (*P* < 0.01)

To assess discriminative AI performance, the test and independent datasets were stratified by oocyte age only, to minimize the confounding effect of including donor oocyte recipient cycles. A comparison of both datasets revealed consistent and significant differences in AI scores within each age category (*P* < 0.01). Furthermore, mean AI scores were significantly higher for FH + embryos than for FH- embryos within each age category (Fig. 1).

When considering all embryos, the test dataset ROC-AUC was 0.762 [95% CI: 0.737–0.786], and the independent dataset ROC-AUC was 0.709 [95% CI: 0.683–0.737]. When considering only embryos with known transfer outcomes only, the test dataset ROC-AUC was 0.630 [95% CI: 0.596–0.665], and the independent dataset ROC-AUC was 0.659 [95% CI: 0.625–0.694]. Though AUC values alone cannot be used to measure and compare the strength of predictive AI models (see Discussion), it is important to note that these values fall within range of other reported AI pregnancy prediction tools [24].

### Correlation with conventional annotations

**Fig. 2:**
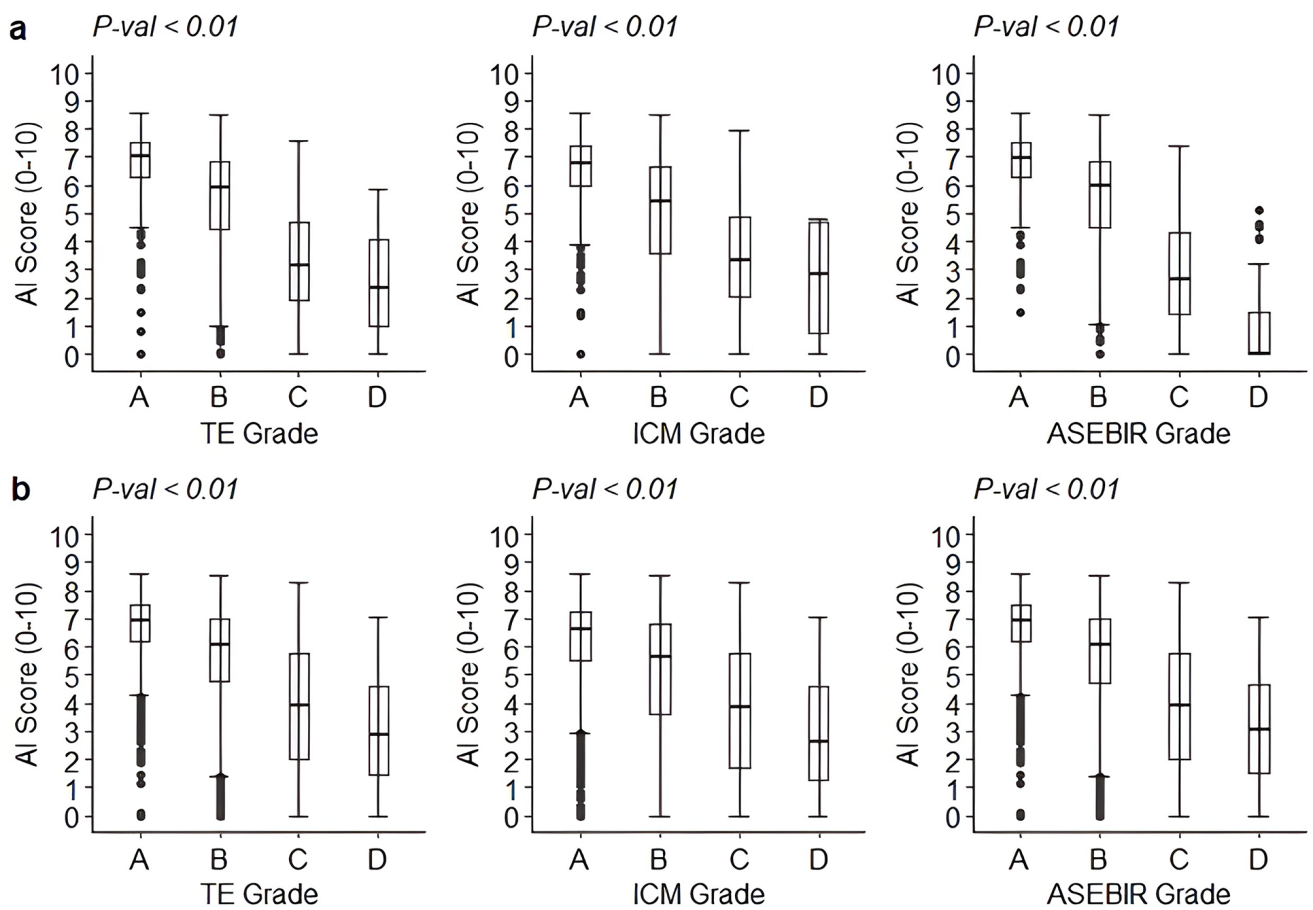
Evaluating the associations between AI scores and annotated trophectoderm (TE) morphology quality grades, inner cell mass (ICM) morphology quality grades, and overall ASEBIR quality grades for the test dataset (**a**) and independent dataset (**b**), respectively

The predictive value of embryo morphologic quality grading has long served as a proxy indicator for clinical pregnancy success. Therefore, we analyzed the correlation between AI scores and conventional morphologic parameters of embryo quality annotated in the data: TE morphology grade, ICM morphology grade, and final ASEBIR grade. Supplemental Table 4 presents the full distribution of annotations and mean AI score values relative to each morphologic parameter for both datasets. Supplemental Table 5 shows the distribution of morphologic annotations along with FH outcomes for each morphologic parameter. The proportion of FH + embryos increased appropriately with ascending ICM morphology, TE morphology, and final ASEBIR grade. Likewise, significant positive associations were observed between increasing AI scores and improving TE quality, ICM quality, and ASEBIR grade, respectively (Fig. 2). AI scores for annotated data: TE quality grade-A 7.67 ± 1.25 versus grade-B 6.61 ± 1.80 versus grade-C 4.76 ± 2.27, *P* < 0.01; ICM quality grade-A 7.25 ± 1.53 versus grade-B 6.05 ± 2.15 versus grade-C 4.68 ± 2.32, *P* < 0.01; ASEBIR grade-A 7.68 ± 1.22 versus grade-B 6.63 ± 1.79 versus grade-C 4.68 ± 2.27, *P* < 0.01. This analysis demonstrated a linear relationship between ascending embryo quality and AI scores, as well as significant AI score differences between each quality group (A versus B versus C, *P* < 0.05) for all three evaluated morphology parameters. Though box plot analysis of AI score distributions relative to each morphology parameter showed generally wide score distributions with long tail lengths (Fig. 2), such presentation was expected due to the inherent inter- and intra-clinic variability in morphologic annotations used as ground truth for this analysis [6, 35, 36].

To assess the AI’s capacity to distinguish between embryo qualities and its association with FH rates, scores were stratified into four brackets, labeled G1, G2, G3, and G4 (G1 reflects the lowest score bracket and G4 reflects the highest score bracket). Brackets were assigned as follows: G1 for scores < 4, G2 for scores 4.0–6.0, G3 for scores 6.1–7.5, and G4 for scores > 7.5. The proportion of FH + embryos progressively increased with ascending score brackets in both datasets (Supplemental Fig. 1), showing a trend of increasing OR. OR was highest in the top G4 bracket (test dataset G4 score > = 7.5: OR 3.837 [95% CI: 2.664–5.527]; independent dataset G4 score > = 7.5: OR 4.009 [95% CI: 2.841–5.656]), and lowest in the G1 bracket (test dataset G1 score < 4.0: OR 0.399 [95% CI :0.295–0.539]; independent dataset G1 score: <4.0: OR 0.455 [95% CI: 0.346–0.597]) (Supplemental Table 6). AI score brackets G2, G3, and G4 appropriately showed OR values above 1.0 (*P* < 0.05), demonstrating significant and linear associations with FH likelihood. AI score bracket G1 (score < 4) showed an OR value below 1.0 (*P* < 0.01) in both datasets, as expected, since low AI scores (< 4.0) are not intended to associate with FH + likelihood. The difference in FH rates between AI score brackets was also significant (*P* < 0.01).

To confer more granularity to the AI scalar rating system, a fifth AI score bracket was recognized for the independent dataset, labeled G5 (score > = 8.9; *N* = 55 embryos). Since known transfer outcomes were available for these embryos, the FH rates for ASEBIR grade-A embryos, G4 scored embryos, and G5 scored embryos were compared. ASEBIR grade-A embryos had an FH rate of 68.65%; G4 scored embryos had an FH rate of 64.56%; and G5 scored embryos had an FH rate of 74.54%. A non-statistical comparison of these values (due to small sample size and dependency between group data) reflected a superior FH rate for embryos scored > = 8.9 (G5 scored embryos) compared to embryos scored > = 7.5 (G4 scored embryos), even though embryos in both categories were considered morphologically top-quality.

## Discussion

This study is notably the first to evaluate the performance of this AI model across 10 clinics in 9 countries. By using multiple representative datasets, we followed a comprehensive methodology for the development and validation of the AI model, ensuring its reliability in evaluating blastocyst-stage embryos.

The test dataset had no overlap in patients or embryos with the training dataset but was obtained from the same clinic cohort. In contrast, the independent dataset was derived from entirely new, ‘unseen’ clinic cohorts, representing distinct demographic and clinical practice profiles not previously analyzed by the AI model. This approach is integral to our validation, since the AI is intended to be applicable to clinics excluded from the training process. Additionally, we did not artificially limit the datasets (such as by cycle type, stimulation protocol, or oocyte donor type), as the AI is also intended to be utilized in a variety of clinical settings without predefined selection criteria. This training and study design allowed us to interrogate potential general biases that could arise from sampling the same clinic cohort for AI training.

The results from the test and independent datasets show a clear trend of increasing AI scores correlating with higher-quality morphologic parameters and improved clinical outcomes. Notably, embryos categorized in the highest AI score bracket (G4, score ≥ 7.5) exhibited the highest odds ratios (OR) for achieving a positive fetal heartbeat (FH), whereas embryos in the lowest bracket (G1, score < 4.0) had the lowest ORs. This consistent performance across diverse datasets underscores the AI model’s potential utility in clinical settings, where a reliable evaluation of embryo quality is critical for optimizing pregnancy outcomes.

The AI model’s potential to discern disparities in FH + rates among morphologically similar embryos further reflects its clinical utility. By identifying subtle differences among embryos that might appear similar under conventional grading, the AI model can enhance conventional embryo evaluation, enabling a more accurate ranking of embryos deemed suitable for transfer. This is especially valuable in single embryo transfer (SET) cycles, where selecting the most viable embryo is critical for achieving a successful pregnancy.

Importantly, the continuous scoring system employed by the AI model offers distinct advantages over conventional discrete grading systems [37]. By providing a continuous assessment of embryo quality, the AI model reduces the subjectivity and variability associated with predefined categories of embryo quality. This is particularly important in the context of inter- and intra-observer variability, where different embryologists might assess the same embryo differently [37].

It is important to frame the context of this study. Evaluating techniques for embryo selection and assessing their relative performance is challenging, primarily because the pregnancy outcomes for each individual embryo in a patient cohort are generally unknown. Extended culture of embryos to the blastocyst stage serves as a ““self-selection” mechanism, correlating with live birth rates [38, 39], and reflects the competence of earlier cleavage-stage embryos [40]. Thus, ranking and selecting blastocysts in the clinic is an important, though imperfect, process that impacts IVF outcomes, such as the number of embryos frozen [41] and transfer order [42]. However, this process is highly variable and subjective [36, 43] and is unlikely to significantly influence the cumulative live birth rate [44]. AI assistance could therefore improve the precision and consistency of blastocyst-stage embryo evaluation [37]. Indeed, multiple blastocyst-stage embryo evaluation tools have been reported for pregnancy prediction [11, 24, 45], providing users with diverse options. This diversity promotes innovation by encouraging competition and continuous AI improvement, while also providing user flexibility. In this manuscript, we focus exclusively on the performance of the AIVF Day-5 AI model, presenting it as a promising option within this landscape.

### Limitations

The main limitation of the present analysis is its retrospective design. While this study provides evidence of the AI’s effectiveness, factors such as population characteristics, model biases, dataset diversity, label distribution, and clinic-specific success rates when integrated into prospective clinical workflows can directly influence performance metrics like accuracy and AUC [24]. A robust prospective analysis would also consider potential clinical confounders, including culture conditions (culture medium, O2 concentration), uterine environment, IVF cycle characteristics (fertilization method, serum hormone levels, body mass index, endometrial preparation protocol, diagnosis), ploidy status, and variable data annotation. This is particularly important, as emerging literature from prospective randomized controlled trials suggests that time-lapse selection with AI does not outperform rigorous morphological evaluation for embryo selection [46, 47]. Ongoing evaluation through prospective randomized trials is essential to eliminate selection bias, control for influencing variables, ensure randomization, and validate AI’s superiority over clinical teams [24].

The AUC is widely used to evaluate performance in embryo evaluation AI models, and we adopt it in our study to align with standard practices. However, its application to embryo ranking is inherently limited [24]. While a high AUC is often interpreted as an indicator of good pregnancy prediction accuracy, AUC strictly quantifies the probability that a randomly selected positive label (clinical pregnancy) is assigned a higher predicted score than a randomly selected negative label (pregnancy failure) within the dataset [24]. Consequently, AUC cannot directly assess the model’s ranking performance within specific patient cohorts, where many embryos are not transferred, and their outcome labels remain unknown [24]. This lack of ground truth labels introduces classification uncertainty, potentially causing overlapping predicted scores for positive and negative pregnancy outcomes.

Furthermore, the influence of AI on embryologist decision-making and the potential biases introduced by AI-assisted evaluations warrants further investigation. Currently, there’s a lack of formal studies quantifying how AI, whether used as an adjunctive tool or stand-alone device, influences embryologist decision-making and subsequent reproductive outcomes in real-time settings. It has been demonstrated that exposure to AI scores, either before or after conventional annotation and selection procedures, could sway and improve relative selection decisions among embryologists [21]. Future studies should focus on quantifying the impact of AI on reproductive outcomes and developing methodologies to ensure the objective and ethical use of AI in reproductive medicine.

## Conclusion

In conclusion, this study presents a validated AI model that offers a reliable and consistent tool for embryo evaluation across multiple clinical settings. The model’s consistent performance across diverse datasets, its continuous scoring system, and its capacity to assist conventional embryo assessment methods make it a promising adjunct to existing IVF practices. Continued research and prospective clinical trials will be essential for fully integrating AI into the IVF laboratory, ultimately improving the efficiency and outcomes of assisted reproductive technologies.

## Electronic supplementary material

Below is the link to the electronic supplementary material.

**Supplementary Material 1**: **Supplemental Fig. 1.** All embryos in the test dataset (red) and independent dataset (blue) were assigned an AI score and classified into ascending score brackets (G1-G4) (X-axis). The fetal heartbeat (FH) rate (Y-axis) was calculated for each score bracket to show ascending rates. **Supplemental Fig. 2**. The time-lapse sequence is fed into the blastulation model, which identifies the presence and timing of blastulation to extract images corresponding to the blastocyst stage. A segmentation network is applied to each frame, with images preprocessed by cropping around the center and resizing to a resolution of 224 × 224 pixels. The core classifier model processes the preprocessed frames, generating a vector of scores based on predictions for positive/negative fetal heartbeat and discard/usable embryo classification for each frame. This vector, along with the time of blastulation and measurements from the segmentation network, is input into the heuristic model to compute a scalar rating between 1 and 9.9. **Supplemental Table 1.** Characteristics of the full dataset used for AI model training and validation. **Supplemental Table 2.** Distribution of data characteristics across the test and independent datasets. **Supplemental Table 3.** Single embryo transfer (SET) characteristics across the test and independent datasets. **Supplemental Table 4.** Distribution of annotations and mean AI scores are shown for three evaluated parameters of embryo morphology quality: ASEBIR grade, inner cell mass (ICM), and trophectoderm (TE). **Supplemental Table 5.** Distribution of annotations and fetal heartbeat (FH) data are shown for three evaluated parameters of embryo morphology quality: ASEBIR grade, inner cell mass (ICM), and trophectoderm (TE). Annotations for transferred embryos ranged from A (top quality) to C (fair quality). All annotated embryos in the test dataset had known FH outcomes. **Supplemental Table 6.** Stratification of fetal heartbeat (FH) outcomes by AI score bracket (G1-G4). Odds ratio (OR) and *P*-values for the associations between AI scores and fetal heartbeat (FH) rate is shown.

## Data Availability

The full code used for data analyses is available on request from the corresponding author. Restrictions apply to the availability of the original data (including embryo videos and patient data), which was used under third-party restrictions outlined by the signed Software Licensing agreement between each clinic and AIVF.

## References

[1] Gardner DK, Meseguer M, Rubio C, Treff NR. Diagnosis of human preimplantation embryo viability. Hum Reprod Update. 2015;21:727–47. 25567750 10.1093/humupd/dmu064

[2] Van Royen E, Mangelschots K, De Neubourg D, Valkenburg M, Van de Meerssche M, Ryckaert G, et al. Characterization of a top quality embryo, a step towards single-embryo transfer. Hum Reprod. 1999;14:2345–9. 10469708 10.1093/humrep/14.9.2345

[3] Rhenman A, Berglund L, Brodin T, Olovsson M, Milton K, Hadziosmanovic N, et al. Which set of embryo variables is most predictive for live birth? A prospective study in 6252 single embryo transfers to construct an embryo score for the ranking and selection of embryos. Hum Reprod. 2015;30:28–36. 25376459 10.1093/humrep/deu295

[4] VerMilyea MD, Tan L, Anthony JT, Conaghan J, Ivani K, Gvakharia M, et al. Computer-automated time-lapse analysis results correlate with embryo implantation and clinical pregnancy: a blinded, multi-centre study. Reprod Biomed Online. 2014;29:729–36. 25444507 10.1016/j.rbmo.2014.09.005 PMC4575500

[5] Diamond MP, Suraj V, Behnke EJ, Yang X, Angle MJ, Lambe-Steinmiller JC, et al. Using the Eeva Test adjunctively to traditional day 3 morphology is informative for consistent embryo assessment within a panel of embryologists with diverse experience. J Assist Reprod Genet. 2015;32:61–8. 25331427 10.1007/s10815-014-0366-1 PMC4294872

[6] Bormann CL, Thirumalaraju P, Kanakasabapathy MK, Kandula H, Souter I, Dimitriadis I, et al. Consistency and objectivity of automated embryo assessments using deep neural networks. Fertil Steril. 2020;113:781–e71. 32228880 10.1016/j.fertnstert.2019.12.004 PMC7583085

[7] Martinez-Granados L, Serrano M, Gonzalez-Utor A, Ortiz N, Badajoz V, Olaya E, et al. Inter-laboratory agreement on embryo classification and clinical decision: conventional morphological assessment vs. time lapse. PLoS ONE. 2017;12:e0183328. 28841654 10.1371/journal.pone.0183328 PMC5571938

[8] Cavalera F, Zanoni M, Merico V, Bui TTH, Belli M, Fassina L, et al. A neural network-based identification of developmentally competent or incompetent mouse fully-grown oocytes. J Vis Exp; 2018. 10.3791/56668 PMC5931424 29553524

[9] Javadi S, Mirroshandel SA. A novel deep learning method for automatic assessment of human sperm images. Comput Biol Med. 2019;109:182–94. 31059902 10.1016/j.compbiomed.2019.04.030

[10] Feyeux M, Reignier A, Mocaer M, Lammers J, Meistermann D, Barriere P, et al. Development of automated annotation software for human embryo morphokinetics. Hum Reprod. 2020;35:557–64. 32163566 10.1093/humrep/deaa001

[11] VerMilyea M, Hall JMM, Diakiw SM, Johnston A, Nguyen T, Perugini D, et al. Development of an artificial intelligence-based assessment model for prediction of embryo viability using static images captured by optical light microscopy during IVF. Hum Reprod. 2020;35:770–84. 32240301 10.1093/humrep/deaa013 PMC7192535

[12] Khosravi P, Kazemi E, Zhan Q, Malmsten JE, Toschi M, Zisimopoulos P, et al. Deep learning enables robust assessment and selection of human blastocysts after in vitro fertilization. NPJ Digit Med. 2019;2:21. 31304368 10.1038/s41746-019-0096-y PMC6550169

[13] Tran D, Cooke S, Illingworth PJ, Gardner DK. Deep learning as a predictive tool for fetal heart pregnancy following time-lapse incubation and blastocyst transfer. Hum Reprod. 2019;34:1011–8. 31111884 10.1093/humrep/dez064 PMC6554189

[14] Bormann CL, Kanakasabapathy MK, Thirumalaraju P, Gupta R, Pooniwala R, Kandula H et al. Performance of a deep learning based neural network in the selection of human blastocysts for implantation. Elife 2020;9. 10.7554/eLife.55301 PMC7527234 32930094

[15] Chavez-Badiola A, Flores-Saiffe-Farias A, Mendizabal-Ruiz G, Drakeley AJ, Cohen J. Embryo Ranking Intelligent classification algorithm (ERICA): artificial intelligence clinical assistant predicting embryo ploidy and implantation. Reprod Biomed Online. 2020;41:585–93. 32843306 10.1016/j.rbmo.2020.07.003

[16] Lee CI, Su YR, Chen CH, Chang TA, Kuo EE, Zheng WL, et al. End-to-end deep learning for recognition of ploidy status using time-lapse videos. J Assist Reprod Genet. 2021;38:1655–63. 34021832 10.1007/s10815-021-02228-8 PMC8324635

[17] Barnes J, Brendel M, Gao VR, Rajendran S, Kim J, Li Q, et al. A non-invasive artificial intelligence approach for the prediction of human blastocyst ploidy: a retrospective model development and validation study. Lancet Digit Health. 2023;5:e28–40. 36543475 10.1016/S2589-7500(22)00213-8 PMC10193126

[18] Bamford T, Easter C, Montgomery S, Smith R, Dhillon-Smith RK, Barrie A, et al. A comparison of 12 machine learning models developed to predict ploidy, using a morphokinetic meta-dataset of 8147 embryos. Hum Reprod. 2023;38:569–81. 36825452 10.1093/humrep/dead034

[19] Diakiw SM, Hall JMM, VerMilyea MD, Amin J, Aizpurua J, Giardini L, et al. Development of an artificial intelligence model for predicting the likelihood of human embryo euploidy based on blastocyst images from multiple imaging systems during IVF. Hum Reprod. 2022;37:1746–59. 35674312 10.1093/humrep/deac131 PMC9340116

[20] Vagios S, James KE, Sacha CR, Hsu JY, Dimitriadis I, Bormann CL, et al. A patient-specific model combining antimullerian hormone and body mass index as a predictor of polycystic ovary syndrome and other oligo-anovulation disorders. Fertil Steril. 2021;115:229–37. 33077236 10.1016/j.fertnstert.2020.07.023

[21] Fitz VW, Kanakasabapathy MK, Thirumalaraju P, Kandula H, Ramirez LB, Boehnlein L, et al. Should there be an AI in TEAM? Embryologists selection of high implantation potential embryos improves with the aid of an artificial intelligence algorithm. J Assist Reprod Genet. 2021;38:2663–70. 34535847 10.1007/s10815-021-02318-7 PMC8581077

[22] Letterie G, Mac Donald A. Artificial intelligence in in vitro fertilization: a computer decision support system for day-to-day management of ovarian stimulation during in vitro fertilization. Fertil Steril. 2020;114:1026–31. 33012555 10.1016/j.fertnstert.2020.06.006

[23] Curchoe CL, Flores-Saiffe Farias A, Mendizabal-Ruiz G, Chavez-Badiola A. Evaluating predictive models in reproductive medicine. Fertil Steril. 2020;114:921–6. 33160514 10.1016/j.fertnstert.2020.09.159

[24] Kragh MF, Karstoft H. Embryo selection with artificial intelligence: how to evaluate and compare methods. J Assist Reprod Genet. 2021;38(7):1675–89. 10.1007/s10815-021-02254-6 PMC8324599 34173914

[25] Letterie G. Three ways of knowing: the integration of clinical expertise, evidence-based medicine, and artificial intelligence in assisted reproductive technologies. J Assist Reprod Genet 2021. 10.1007/s10815-021-02159-4 PMC8324699 33870475

[26] Dimitriadis I, Zaninovic N, Badiola AC, Bormann CL. Artificial intelligence in the embryology laboratory: a review. Reprod Biomed Online. 2022;44:435–48. 35027326 10.1016/j.rbmo.2021.11.003

[27] Curchoe CL, Malmsten J, Bormann C, Shafiee H, Flores-Saiffe Farias A, Mendizabal G, et al. Predictive modeling in reproductive medicine: where will the future of artificial intelligence research take us? Fertil Steril. 2020;114:934–40. 33160516 10.1016/j.fertnstert.2020.10.040

[28] Zaninovic N, Rosenwaks Z. Artificial intelligence in human in vitro fertilization and embryology. Fertil Steril. 2020;114:914–20. 33160513 10.1016/j.fertnstert.2020.09.157

[29] Sfakianoudis K, Maziotis E, Grigoriadis S, Pantou A, Kokkini G, Trypidi A et al. Reporting on the Value of Artificial Intelligence in Predicting the optimal embryo for transfer: a systematic review including data synthesis. Biomedicines 2022;10. 10.3390/biomedicines10030697 PMC8945147 35327499

[30] Gardner DK. The way to improve ART outcomes is to introduce more technologies in the laboratory. Reprod Biomed Online. 2022;44:389–92. 34911663 10.1016/j.rbmo.2021.10.021

[31] Harper J, Magli MC, Lundin K, Barratt CL, Brison D. When and how should new technology be introduced into the IVF laboratory? Hum Reprod. 2012;27:303–13. 22166806 10.1093/humrep/der414

[32] Gardner DK, Lane M, Stevens J, Schlenker T, Schoolcraft WB. Blastocyst score affects implantation and pregnancy outcome: towards a single blastocyst transfer. Fertil Steril. 2000;73:1155–8. 10856474 10.1016/s0015-0282(00)00518-5

[33] Garcia-Belda A, Cairo O, Martinez-Moro A, Cuadros M, Pons MC, de Mendoza MVH, et al. Considerations for future modification of the Association for the Study of Reproductive Biology embryo grading system incorporating time-lapse observations. Reprod Biomed Online. 2024;48:103570. 37952277 10.1016/j.rbmo.2023.103570

[34] Irene Cuevas Saiz MCPG, Vargas MC, Mendive AD. Natalia Rives Enedáguila, Marta Moragas Solanes, Beatriz Carrasco Canal, José Teruel López, Ana Busquets Bonet, Mª Victoria Hurtado De Mendoza Acosta. The Embryology Interest Group: updating ASEBIR’s morphological scoring system for early embryos, morulae and blastocysts. Med Reproductiva Y Embriología Clínica. 2018;5:42–54.

[35] Baxter Bendus AE, Mayer JF, Shipley SK, Catherino WH. Interobserver and intraobserver variation in day 3 embryo grading. Fertil Steril. 2006;86:1608–15. 17074349 10.1016/j.fertnstert.2006.05.037

[36] Curchoe CL, Bormann C, Hammond E, Salter S, Timlin C, Williams LB, et al. Assuring quality in assisted reproduction laboratories: assessing the performance of ART compass - a digital art staff management platform. J Assist Reprod Genet. 2023;40:265–78. 36637586 10.1007/s10815-023-02713-2 PMC9935773

[37] Kragh MF, Rimestad J, Berntsen J, Karstoft H. Automatic grading of human blastocysts from time-lapse imaging. Comput Biol Med. 2019;115:103494. 31630027 10.1016/j.compbiomed.2019.103494

[38] Glujovsky D, Quinteiro Retamar AM, Alvarez Sedo CR, Ciapponi A, Cornelisse S, Blake D. Cleavage-stage versus blastocyst-stage embryo transfer in assisted reproductive technology. Cochrane Database Syst Rev. 2022;5:CD002118. 35588094 10.1002/14651858.CD002118.pub6 PMC9119424

[39] Kovacs P, Sun S, Lu Y, Romanski P, Lindheim SR. Benefits of blastocyst transfer with at least three good-quality cleavage-stage embryos in women of advanced maternal age: a retrospective analysis. J Obstet Gynaecol Can 2023:102233. 10.1016/j.jogc.2023.102233 37820927

[40] Liu Z, Cai J, Liu L, Ouyang L, Chen J, Yang C, et al. Does cleavage stage morphology increase the discriminatory power of prediction in blastocyst transfer outcome? J Assist Reprod Genet. 2024;41:347–58. 38040894 10.1007/s10815-023-02997-4 PMC10894791

[41] Song J, Duan C, Cai W, Xu J. Predictive value of the number of frozen blastocysts in live birth rates of the transferred fresh embryos. J Ovarian Res. 2021;14:83. 34174916 10.1186/s13048-021-00838-5 PMC8236141

[42] Zou H, Kemper JM, Hammond ER, Xu F, Liu G, Xue L, et al. Blastocyst quality and reproductive and perinatal outcomes: a multinational multicentre observational study. Hum Reprod. 2023;38:2391–9. 37877423 10.1093/humrep/dead212 PMC10694400

[43] Racowsky C, Vernon M, Mayer J, Ball GD, Behr B, Pomeroy KO, et al. Standardization of grading embryo morphology. Fertil Steril. 2010;94:1152–3. 20580357 10.1016/j.fertnstert.2010.05.042

[44] Mastenbroek S, van der Veen F, Aflatoonian A, Shapiro B, Bossuyt P, Repping S. Embryo selection in IVF. Hum Reprod. 2011;26:964–6. 21372045 10.1093/humrep/der050

[45] Berntsen J, Rimestad J, Lassen JT, Tran D, Kragh MF. Robust and generalizable embryo selection based on artificial intelligence and time-lapse image sequences. PLoS ONE. 2022;17(2):e0262661. 35108306 10.1371/journal.pone.0262661 PMC8809568

[46] Illingworth PJ, Venetis C, Gardner DK, et al. Deep learning versus manual morphology-based embryo selection in IVF: a randomized, double-blind noninferiority trial. Nat Med. 2024;30(12):3114–20. 39122964 10.1038/s41591-024-03166-5 PMC11564097

[47] Ahlström A, Lundin K, Lind AK, Gunnarsson K, Westlander G, Park H, et al. A double-blind randomized controlled trial investigating a time-lapse algorithm for selecting Day 5 blastocysts for transfer. Hum Reprod. 2022;37(4):708–17. 35143661 10.1093/humrep/deac020 PMC9383441

