# Supplementary material for: Application of a methodological framework for the development and multicenter validation of reliable artificial intelligence in embryo evaluation
Source: Reprod Biol Endocrinol. 2025 Jan 31;23:16. doi: 10.1186/s12958-025-01351-w (PMC11783712; doi:10.1186/s12958-025-01351-w)
Supplement: Supplementary file 1 — Supplementary Material 1: Supplemental Fig. 1. All embryos in the test dataset (red) and independent dataset (blue) were assigned an AI score and classified into ascending score brackets (G1-G4) (X-axis). The fetal heartbeat (FH) rate (Y-axis) was calculated for each score bracket to show ascending rates. Supplemental Fig. 2. The time-lapse sequence is fed into the blastulation model, which identifies the presence and timing of blastulation to extract images corresponding to the blastocyst stage. A segmentation network is applied to each frame, with images preprocessed by cropping around the center and resizing to a resolution of 224 × 224 pixels. The core classifier model processes the preprocessed frames, generating a vector of scores based on predictions for positive/negative fetal heartbeat and discard/usable embryo classification for each frame. This vector, along with the time of blastulation and measurements from the segmentation network, is input into the heuristic model to compute a scalar rating between 1 and 9.9. Supplemental Table 1. Characteristics of the full dataset used for AI model training and validation. Supplemental Table 2. Distribution of data characteristics across the test and independent datasets. Supplemental Table 3. Single embryo transfer (SET) characteristics across the test and independent datasets. Supplemental Table 4. Distribution of annotations and mean AI scores are shown for three evaluated parameters of embryo morphology quality: ASEBIR grade, inner cell mass (ICM), and trophectoderm (TE). Supplemental Table 5. Distribution of annotations and fetal heartbeat (FH) data are shown for three evaluated parameters of embryo morphology quality: ASEBIR grade, inner cell mass (ICM), and trophectoderm (TE). Annotations for transferred embryos ranged from A (top quality) to C (fair quality). All annotated embryos in the test dataset had known FH outcomes. Supplemental Table 6. Stratification of fetal heartbeat (FH) outcomes by AI score brac [file 12958_2025_1351_MOESM1_ESM.docx]

**Supplemental figure 1.** All embryos in the test dataset (red) and independent dataset (blue) were assigned an AI score and classified into ascending score brackets (G1-G4) (X-axis). The fetal heartbeat (FH) rate (Y-axis) was calculated for each score bracket to show ascending rates


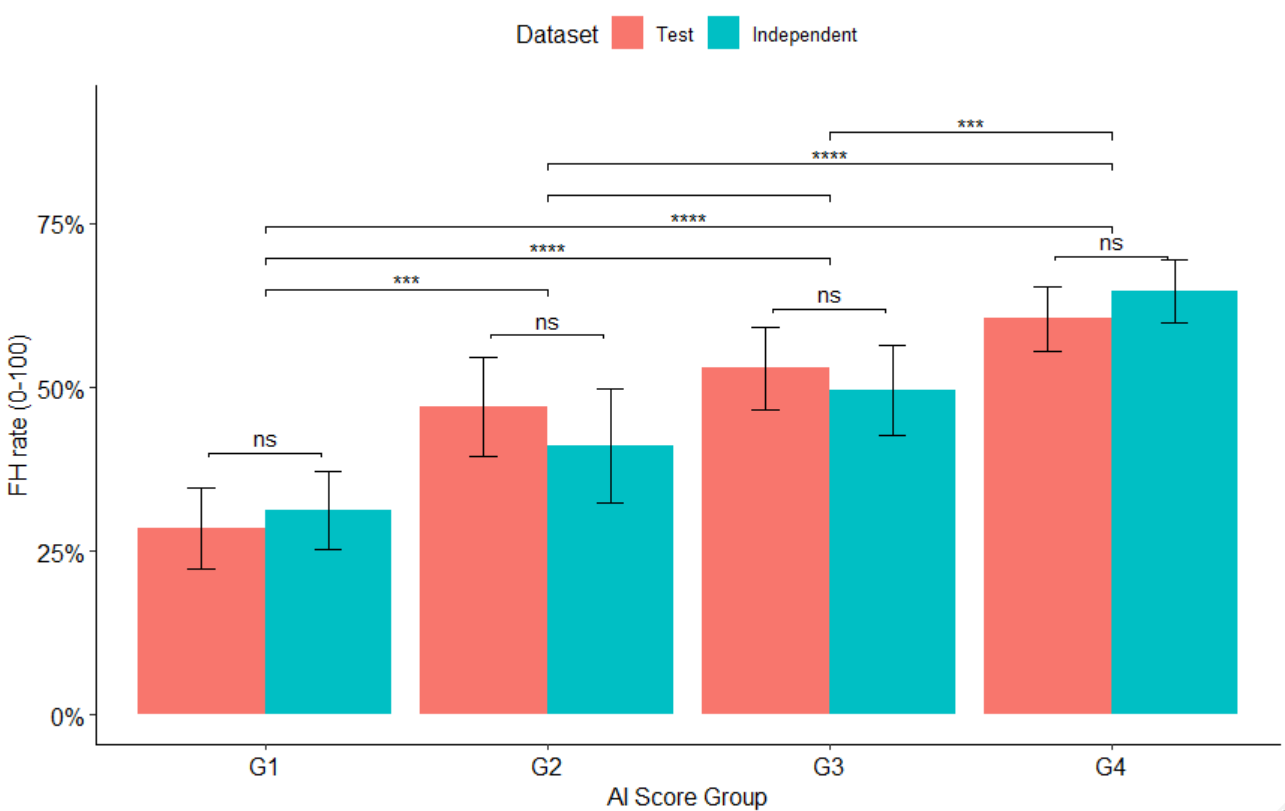


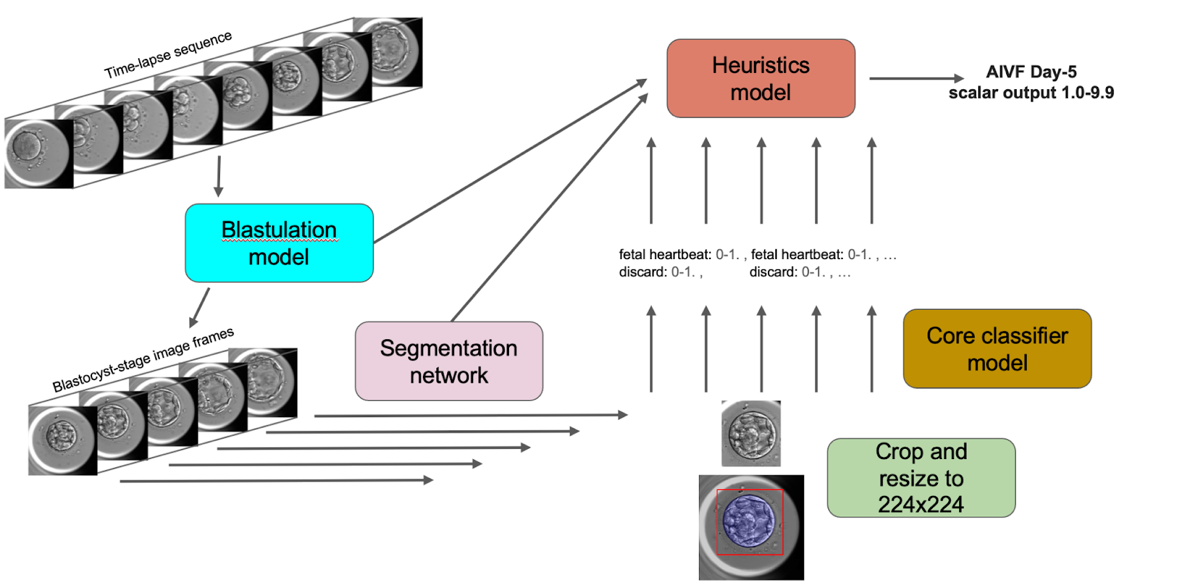
**Supplemental Figure 2.** The time-lapse sequence is fed into the blastulation model, which identifies the presence and timing of blastulation to extract images corresponding to the blastocyst stage. A segmentation network is applied to each frame, with images preprocessed by cropping around the center and resizing to a resolution of 224x224 pixels. The core classifier model processes the preprocessed frames, generating a vector of scores based on predictions for positive/negative fetal heartbeat and discard/usable embryo classification for each frame. This vector, along with the time of blastulation and measurements from the segmentation network, is input into the heuristic model to compute a scalar rating between 1 and 9.9

**Supplemental Table 1.** Characteristics of the full dataset used for AI model training and validation

| **Characteristics** | **Train and validation dataset** |
| --- | --- |
| Total embryo count (n=embryos) | 16,935 |
| known transfer outcomes (n=embryos) | 9,810 |
| FH+ embryos (n=embryos) | 4,812 |
| FH- embryos (n=embryos) | 4,998 |
| FH rate (%) | 49 |
| Discarded (n=embryos) | 7,125 |
| Fresh transferred (n=embryos) | 5,577 |
| Cryopreserved-thawed transferred (n=embryos) | 4,233 |
| Maternal (uterus) age (mean ± SD, years) | 38.09 ± 5.26 |
| Oocyte age (mean ± SD, years) | 30.82 ± 6.75 |

**Supplemental Table 2.** Distribution of data characteristics across the test and independent datasets

| **Characteristics** | **Test dataset** | **Independent dataset** |
| --- | --- | --- |
| Total embryo count (n=embryos) | 1,708 | 7,445 |
| After data exclusion (n=embryos) | 1,708 | 6,246 |
| Transferred (n=embryos) | 1,070 | 1,493 |
| Single embryo transferred (n=embryos) | 997 | 1,168 |
| Double embryo transferred (n=embryos) | 64 | 316 |
| Triple embryo transferred (n=embryos) | 9 | - |
| Cryopreserved (n=embryos) | 25 | 2,985 |
| Discarded (n=embryos) | 596 | 992 |
| Unknown clinical fate (n=embryos) | 17 | 776 |
| Unknown outcome after transfer (n=embryos) | - | 9 |
| Annotated (n=embryos) | 1,358 | 5,734 |
| Maternal (uterus) age (mean ± SD, years) | 38.76 ± 5.21 | 37.00 ± 4.88 |
| Oocyte age (mean ± SD, years) | 30.20 ± 6.96 | 35.79 ± 4.12 |

**Supplemental Table 3.** Single embryo transfer (SET) characteristics across the test and independent datasets

| **Characteristics** | **Test dataset** | | **Independent Dataset** | |
| --- | --- | --- | --- | --- |
|  | **Autologous** | **Donor oocyte recipient** | **Autologous** | **Donor oocyte recipient** |
| Single transferred embryos (n= embryos) | 582 | 415 | 936 | 232 |
| Known transfer outcomes (n= embryos) | 582 | 415 | 791 | 171 |
| Positive FH outcome (n=embryos) | 240 | 256 | 368 | 111 |
| Maternal (uterus) age  (mean ± SD, years) | 35.2 ± 4.56 | 41.2 ± 4.26 | 35.4 ± 3.85 | 41.8 ± 4.40 |
| Oocyte age  (mean ± SD, years) | 35.2 ± 4.56 | 25.4 ± 4.34 | 35.4 ± 3.85 | 25.4 ± 4.47 |
| FH rate (%)  [95% CI] | 41.23 [37.20-45.20] | 61.68 [57.00-66.40] | 46.52 [43.00-50.00] | 64.91 [57.70-72.10] |

**Supplemental Table 4.**  Distribution of annotations and mean AI scores are shown for three evaluated parameters of embryo morphology quality: ASEBIR grade, inner cell mass (ICM), and trophectoderm (TE)

| **Morphology parameter** |  | **Test Dataset** | | **Independent Dataset** | | **Combined Data** | |
| --- | --- | --- | --- | --- | --- | --- | --- |
|  | **Annotation** | **Embryo count (n)** | **AI score (mean ± SD)** | **Embryo count** | **AI score (mean ± SD)** | **Embryo count (n)** | **AI score (mean ± SD)** |
| **ASEBIR** | A | 221 | 7.72 ± 1.20 | 882 | 7.67 ± 1.23 | 1103 | 7.68 ± 1.22 |
|  | B | 523 | 6.50 ± 1.81 | 2190 | 6.65 ± 1.78 | 2713 | 6.63 ± 1.79 |
|  | C | 445 | 3.92 ± 1.94 | 2496 | 4.81 ± 2.30 | 2941 | 4.68 ± 2.27 |
|  | D | 49 | 2.01 ± 1.53 | 166 | 4.10 ± 1.94 | 215 | 3.62 ± 2.05 |
|  | E | 120 | 2.04 ± +1.81 | - | - | 120 | 2.04 ± +1.81 |
| **ICM** | A | 317 | 7.45 ± 1.42 | 1299 | 7.21 ± 1.56 | 1616 | 7.25 ± 1.53 |
|  | B | 534 | 6.00 ± 2.08 | 2674 | 6.06 ± 2.16 | 3208 | 6.05 ± 2.15 |
|  | C | 221 | 4.51 ± 2.01 | 1475 | 4.71 ± 2.36 | 1696 | 4.68 ± 2.32 |
|  | D | 4 | 3.60 ± 2.46 | 73 | 3.98 ± 2.10 | 77 | 3.97 ± 2.10 |
| **TE** | A | 235 | 7.68 ± 1.32 | 882 | 7.67 ± 1.23 | 1117 | 7.67 ± 1.25 |
|  | B | 552 | 6.45 ± 1.87 | 2191 | 6.66 ± 1.78 | 2743 | 6.61 ± 1.80 |
|  | C | 296 | 4.30 ± 1.94 | 2504 | 4.82 ± 2.29 | 2800 | 4.76 ± 2.27 |
|  | D | 17 | 3.47 ± 1.86 | 157 | 4.01 ± 1.91 | 174 | 3.96 ± 1.91 |

**Supplemental Table 5**. Distribution of annotations and fetal heartbeat (FH) data are shown for three evaluated parameters of embryo morphology quality: ASEBIR grade, inner cell mass (ICM), and trophectoderm (TE). Annotations for transferred embryos ranged from A (top quality) to C (fair quality). All annotated embryos in the test dataset had known FH outcomes

|  | | **Test Dataset** | | | **Independent Dataset** | | | |
| --- | --- | --- | --- | --- | --- | --- | --- | --- |
| **Morphology parameter** | **Annotation** | **Embryo count (n)** | **FH+ embryos (n)** | **FH rate (%)**  [95% CI] | **Embryo count (n)** | **Embryos with known FH outcomes** | **FH+ embryos (n)** | **FH rate (%)** [95% CI] |
| **ASEBIR** | A | 207 | 133 | 64.25 [57.70-70.80] | 180 | 134 | 92 | 68.65 [60.70-76.60] |
|  | B | 407 | 231 | 56.75 [51.90-61.60] | 566 | 470 | 264 | 56.17 [51.70-60.70] |
|  | C | 124 | 50 | 40.32 [31.60-49.10] | 205 | 170 | 61 | 35.88 [28.60-43.20] |
| **ICM** | A | 290 | 181 | 62.41 [56.80-68.00] | 289 | 216 | 136 | 62.96 [56.50-69.50] |
|  | B | 381 | 207 | 54.33 [49.30-59.40] | 611 | 530 | 271 | 51.13 [46.90-55.40] |
|  | C | 57 | 25 | 43.85 [30.60-57.10] | 43 | 27 | 7 | 25.92 [8.26-43.60] |
| **TE** | A | 215 | 139 | 64.65 [58.20-71.10] | 180 | 134 | 92 | 68.65 [60.70-76.60] |
|  | B | 430 | 241 | 56.04 [51.30-60.80] | 566 | 470 | 264 | 56.17 [51.70-60.70] |
|  | C | 86 | 35 | 40.69 [30.10-51.30] | 205 | 170 | 61 | 35.88 [28.60-43.20] |

**Supplemental Table 6.** Stratification of fetal heartbeat (FH) outcomes by AI score bracket (G1-G4). Odds ratio (OR) and *P*-values for the associations between AI scores and fetal heartbeat (FH) rate is shown

|  | **Test Dataset** | | | | | **Independent dataset** | | | | |
| --- | --- | --- | --- | --- | --- | --- | --- | --- | --- | --- |
| **AI score**  **bracket** | **Embryo count**  **(n)** | **FH+ embryos**  **(n)** | **FH rate (%)**  **[95% CI]** | **OR**  **[95% CI]** | ***P*-**  **value** | **Embryo count (n)** | **FH+ embryos (n)** | **FH rate (%)**  **[95% CI]** | **OR**  **[95% CI]** | ***P-***  **value** |
| **G4**  **(score>=7.5)** | 382 | 231 | 60.47 [55.40-64.90] | 3.84  [2.66-5.53] | <0.01 | 381 | 246 | 64.56  [59.70-69.40] | 4.01  [2.84-5.66] | <0.01 |
| **G3**  **(>=6 score <7.5)** | 240 | 127 | 52.91 [46.80-59.10] | 2.82  [1.90-4.18] | <0.01 | 208 | 103 | 49.51  [42.70-56.40] | 2.16  [1.47-3.17] | 0.05 |
| **G2**  **(>=4 score <6)** | 168 | 79 | 47.02  [39.10-53.70] | 2.23  [1.45-3.42] | <0.01 | 129 | 53 | 41.08  [32.50-49.70] | 1.53  [0.98-2.39] | <0.01 |
| **G1**  **(score<4)** | 207 | 59 | 28.50  [22.30-34.20] | 0.40  [0.30-0.54] | <0.01 | 240 | 75 | 31.25  [25.30-37.20] | 0.46  [0.35- 0.60] | <0.01 |
